# Apilimod activates the NLRP3 inflammasome through lysosome-mediated mitochondrial damage

**DOI:** 10.3389/fimmu.2023.1128700

**Published:** 2023-06-08

**Authors:** Yingting Hou, Hongbin He, Ming Ma, Rongbin Zhou

**Affiliations:** ^1^ Center for Advanced Interdisciplinary Science and Biomedicine of Institute of Health and Medicine (IHM), Division of Life Sciences and Medicine, University of Science and Technology of China, Hefei, China; ^2^ The Chinese Academy of Sciences (CAS) Key Laboratory of Innate Immunity and Chronic Disease, School of Basic Medical Sciences, Division of Life Sciences and Medicine, University of Science and Technology of China, Hefei, China

**Keywords:** NLRP3 inflammasome, activation, apilimod, lysosome, mitochondria

## Abstract

NLRP3 is an important innate immune sensor that responses to various signals and forms the inflammasome complex, leading to IL-1β secretion and pyroptosis. Lysosomal damage has been implicated in NLRP3 inflammasome activation in response to crystals or particulates, but the mechanism remains unclear. We developed the small molecule library screening and found that apilimod, a lysosomal disruptor, is a selective and potent NLRP3 agonist. Apilimod promotes the NLRP3 inflammasome activation, IL-1β secretion, and pyroptosis. Mechanismically, while the activation of NLRP3 by apilimod is independent of potassium efflux and directly binding, apilimod triggers mitochondrial damage and lysosomal dysfunction. Furthermore, we found that apilimod induces TRPML1-dependent calcium flux in lysosomes, leading to mitochondrial damage and the NLRP3 inflammasome activation. Thus, our results revealed the pro-inflammasome activity of apilimod and the mechanism of calcium-dependent lysosome-mediated NLRP3 inflammasome activation.

## Introduction

The role of NLRP3 in major diseases is emerging, including inflammatory diseases, metabolite disorders and central nervous system diseases (1, 2). Various stimulators could induce the assembly of NLRP3 inflammasome complex, leading to the autoproteolysis of caspase-1, which promotes the maturation of IL-1β (3), IL-18 (4) and GSDMD-mediated pyroptosis (5, 6). These stimulators trigger multiple cellular perturbations, including ion flux (7–10), lysosome disruption (11–13), mitochondrial damage (14–16) and disturbing vesicular trafficking (17), to finally mediate the activation of the NLRP3 inflammasome. However, each hypothesis cannot cover all circumstances. For most NLRP3 agonists, K^+^ efflux is an universal requirement. Recently, it has been found that the TLR7 ligand imiquimod and its related molecule CL097 activate the NLRP3 inflammasome in a potassium-independent manner. Imiquimod and CL097 inhibits mitochondrial complex I to induce ROS production, leading to NLRP3 activation (18, 19). Hence, the mechanism of NLRP3 activation is associated with diverse organelles and remains unclear.

Lysosome, the key degradative compartment of the cell, is a single-membrane cell organelle that contains enzymes to promote the degradation of molecules from host or invading substance (20). Lysosomes get involved with many cell processes (21–23) and play important roles in various diseases (24, 25). Endogenous insolubles (MSU crystals (12), cholesterol crystals (26, 27), amyloid-β aggregates (28) and exogenous particulates (silica and alum (11, 29)), could be phagocytosed by lysosomes and induce the NLRP3 inflammasome activation. The accumulation of engulfed crystals in lysosomes decreases lysosomal pH and promotes the lysosomal membrane’s destabilization, leading to the release of cathepsins (11, 30, 31). It has been demonstrated that cathepsin inhibitors abrogate the NLRP3 inflammasome activation, and multiple cathepsins promote NLRP3 activation, indicating that cathepsins release mediated by lysosomal acidification is involved in the activation of NLRP3 inflammasome (30, 32–36). Cytosolic accumulation of lysosomal proteases and increased permeability of the plasma membrane that causes K^+^ efflux are also found to drive NLRP3 inflammasome activation (37). In addition, there is another mechanism by which phagocytosed crystals or agonists targeting lysosomes cause ion flux to change the cellular osmotic pressure, and activate the NLRP3 inflammasome. Thus, the pivotal signal downstream of lysosomal disruption during NLRP3 inflammasome activation needs to be further investigated.

Apilimod is a selective PIKfyve inhibitor (38), which abrogates the synthesis of IL-12 and IL-23 in patients with Crohn’s disease (CD) (39), rheumatoid arthritis (RA) (40), and psoriasis (41). PIKfyve plays an important role in maintaining lysosome morphology through binding to PtdIns3P with its FYVE domain. Besides, apilimod has anti-viral and anti-tumor effects. In B-cell non-Hodgkin lymphoma, apilimod disrupts lysosomal homeostasis, leading to autophagy dysfunction inhibiting tumor cell proliferation (42, 43). Therefore, apilimod is an effective lysosomal disruptor.

Here, our results showed that apilimod is a newly found selective and potent NLRP3 agonist. Apilimod stimulation promoted the secretion of caspase-1 and IL-1β, which are dependent on NLRP3. Apilimod facilitated calcium release by lysosomes, to induce mitochondrial damage and ROS production. Inhibitors targeting lysosomal acidification, calcium flux, and ROS significantly inhibited apilimod-induced activation of the NLRP3 inflammasome. Taken together, we found that apilimod activates the NLRP3 inflammasome by promoting lysosomal calcium-dependent mitochondrial damage.

## Material and methods

### Mice

C57BL/6J mice were supplied from Shanghai SLAC Laboratory Animal Limited Liability Company (Shanghai, China). *Asc^-/-^* mice were furnished by Dr. Vishva M. Dixit Group. *Casp1^-/-^* mice were furnished by Dr. Richard A. Flavell Group. *Gsdmd^-/-^* mice were furnished by Dr. Shu Zhu Group (University of Science and Technology of China). *Nlrp3^-/-^* mice were furnished by Dr. Jurg Tschopp Group (University of Lausanne). *Aim2^-/-^* mice were furnished by Dr. Bing Sun (Institut Pasteur of Shanghai, Chinese Academy of Sciences). *Mevf^-/^* ^-^ mice were furnished by Dr. Feng Shao (National Institute of Biological Sciences, Beijing). Mice were maintained in a SPF facility under a 12h/12h light/dark cycle (lights on at 07:00 and off at 19:00). All animal experiments were conducted in compliance with the guidelines of the Ethics Committee of University of Science and Technology of China.

### Reagents

Apilimod (No. S6414) was bought from Selleck. Nigericin, MSU, and poly (A/T) were purchased from Sigma-Aldrich. Ultrapure LPS, MitoSOX, MitoTracker, DAPI, Lysotracker Green, MitoTracker Deep Red, MitoTracker Green were acquired from Invitrogen. MnTBAP was bought from Santa Cruz Biotechnology. The C3 toxin was a gift from Dr. Tengchuan Jin (University of Science and Technology of China, Hefei, China). CY-09 was synthesized from Dr. Xianming Deng (Xiamen University, Xiamen, China). MCC950 was bought from Selleck. Fluo-4, AM(HY-101896), ML-SI3 were purchased from MedChemExpress. BAPTA-AM(A1076) was bought from Sigma-Aldrich. Protein G agarose (16–266) was bought from Millipore. Anti-Flag (F2555) antibodies were from Sigma-Aldrich. The anti-mouse NLRP3 (AG-20B-0014) and the anti-mouse caspase-1 (AG-20B-0042) antibodies was from AdipoGen. The anti-mouse IL-1β antibody (AF-401-NA) was obtained from R&D Systems. The anti-β-actin antibody (66009-1-Ig) was from Proteintech Group. The anti-mouse ASC antibody (67824S) was from Cell Signaling Technology. The anti-mouse NEK7 antibody (ab133514) was from Abcam.

### Cell preparation and stimulation

BMDMs were isolated from bone marrow and cultured for 6-7 days in DMEM supplemented with 10% FBS, 1% antibiotics and 20 ng/mL M-CSF.

THP-1 cells were cultured in RPMI-1640 supplemented with 10% FBS and 1% antibiotics.

To activate the NLRP3 inflammasome, BMDMs (5×10^5^ cells/mL) or THP-1 cells (1×10^6^ cells/mL) were planted in 12-well plates, THP-1 cells were treated with PMA (100nM) for one night, and the overnight culture media was replaced with Opti-MEM the following morning. After three hours of priming with 50 ng/mL LPS, BMDMs were stimulated with apilimod or treated with other agents (MCC950, CY09, MnTBAP, Baf-A1, or CA-074-Me) according to the experimental needs for 30 min before the stimulation of apilimod. THP-1 cells were stimulated with apilimod after LPS-priming. Using Lipofectamine 2000, cells were transfected with poly(A/T) (0.5 g/mL) for 2 h. Immunoblotting was used to evaluate supernatants and cell extracts that had been precipitated. Using an LDH Cytotoxicity Assay Kit, LDH release was quantified (Beyotime).

### Confocal microscopy

2 × 10^5^/mL BMDMs were seeded on coverslips (Thermo Fisher Scientific) in 24-well plates overnight. In the following morning, BMDMs were treated with or with not agents (MnTBAP, Baf-A1, or CA-074-Me) according to the experimental needs before stimulated with apilimod. Thirty minutes before the end of stimulation, the above BMDMs were stained with MitoTracker Red (50 nM) or MitoSox (5 μM) or Lysotracker Green (200 nM). Then the supernatants were removed and the cells were washed three times with ice-cold PBS and fixed with 4% PFA in PBS for 15 min. After that, the cells were washed with PBST for three times and stained with DAPI. After 25 min treatment with Lysotracker Green, the cells were stained with Hoechst 33342 for 5 min, then observed under the microscope. The Zeiss LSM 700 was utilized to conduct confocal microscopy analysis.

### ELISA

Supernatants from cell culture and serum were measured for mouse IL-1β, IL-6 or TNF-α, and human IL-1β (R&D Systems) according to the manufacturer’s instructions.

### Western blot

The proteins in cell supernatant were extracted by methanol-chloroform method, then directly diluted by 1.5 × Sample buffer. Denature the samples by boiling the lysates at 95-100˚C for 10 minutes. Polyacrylamide gel electrophoresis was performed, Run the gel for 30 min at 80 V then 1** **h at 120 V. After that, transferred the proteins from the gel to the PVDF membrane under 90 V for 1 hour. The primary antibody was added and PVDF membrane were incubated at 4°C overnight. Next day, incubate the membrane with conjugated secondary antibody in blocking buffer at room temperature for 1 h. Wash the membrane in three washes of PBST, then conduct signal development. The development results were observed with BioRad and analyzed with Image Lab software.

### Flow cytometric analyses

Flow cytometry was used to analyze the following mitochondrial parameters: mitochondrial mass, ROS levels, lysosome acidification and calcium flux. To measure mitochondrial mass, cells were stained with MitoTracker green and MitoTracker deep red at a concentration of 50 nM for 30 min at 37 °C. Mitochondria-associated ROS levels were measured by staining cells with MitoSOX at a concentration of 5 μM for 30 min at 37 °C. Lysosome acidification was measured by staining cells with Lysotracker Green (200 nM) for 30 min at 37 °C. After staining, cells were washed with PBS and re-suspended in cold PBS containing 1% FBS for FACS analysis. To measure calcium flux, incubate the cells with the Fluo-4 AM working solution (2 μM in calcium-free HBSS) for 40 minutes at 37°C in the dark. After incubation, wash the cells with calcium-free HBSS to remove any unbound dye, and analyze by flow cytometry or microscopy.

### Determination of intracellular potassium

BMDMs plated in 6-well plates (1.5×10^6^-2×10^6^ cells/well) was stimulated to active NLRP3 inflammasome and then the cultured mediums were removed and K^+^-free PBS was used to rinse cells for 3 times. 1 mL ultrapure HNO_3_ were added to lyse cells and the samples were boiled at 100 °C for several times until the powders were light yellow. After boiling Then, ddH_2_O was added to dissolve the precipitated products. The samples were measured of potassium by inductively coupled plasma optical emission spectrometry with a PerkinElmer Optima 7300 DV spectrometer.

### ASC oligomerization assay

BMDMs were cultured in 6-well plates and exposed to apilimod for a duration of 2 hours. Following this, the cells were lysed using TBS buffer, which consisted of 50 mM Tris-HCl (purchased from GCRF, China), 150 mM NaCl, and 0.5% Triton X-100 (purchased from Sigma-Aldrich), with a pH of 7.4. Additionally, phosphatase inhibitor (purchased from Roche) and EDTA-free protease inhibitor (purchased from Bimake) were added to the lysate. The cells were incubated on a shaker for 30 minutes at 4 °C and then centrifuged at 6000 × g/4 °C for 15 minutes to remove the supernatant. The pellets were washed twice with TBS buffer, and then resuspended in TBS buffer containing 2 mM fresh disuccinimidyl suberate (DSS, purchased from Thermo Fisher Scientific). The cross-linking was carried out at 37 °C for 30 min with the tubes being flipped every 10 minutes. The samples were then centrifuged at 6000 × g/4 °C for 15 minutes. The cross-linked pellets were resuspended in 25 μl of 3 × SDS loading buffer and boiled at 100 °C for 10 min. Finally, the samples were analyzed by immunoblotting using an anti-ASC antibody.

### Immunoprecipitation

For the endogenous IP assay, BMDMs in 6-well plates were stimulated with apilimod or nigericin and lysed with NP-40 lysis buffer containing complete protease inhibitor. The primary antibodies and Protein G Mag Sepharose were added to the cell lysates and were incubated overnight at 4°C with rolling incubator. The antibody-bound proteins were precipitated by protein G beads and were subjected to immunoblot analysis.

### Drug Affinity Responsive Target Stability (DARTS) assay

The DARTS experiment was performed following a published protocol (39). BMDMs were treated with LPS (50 ng/mL) for 3 hours and subsequently lysed with NP-40 lysis buffer containing complete protease inhibitors. The lysates were then centrifuged at 12,000g for 10 minutes at 4°C and the protein concentration was determined using a Pierce BCA Protein Assay Kit (Beyotime). Apilimod was added to the lysates at specified concentrations and incubated for 1 hour at room temperature with shaking. Pronase (25 ng enzyme per μg of protein, Sigma) was then added to the lysates (8 μg protein lysate per reaction) and incubated for 30 minutes at room temperature. The reaction was stopped by adding 3× SDS loading buffer and the samples were subsequently analyzed by immunoblotting.

### Protein expression and purification

The protocol for expression and purification of His-GFP-NLRP3 has been described previously (44).

### SiRNA-mediated gene silencing in BMDMs

BMDMs (3 × 10^5^ cells/ml) were seeded in 12-well plates. To transfect the cells in each well, Lipofectamine RNAiMAX (Invitrogen) was used following the manufacturer’s instructions, with 50 nM of siRNA (siG6pdx) added to each well.BMDMs were seeded in 12-well plates at a concentration of 3 x 10^5^ cells/ml. To transfect the cells in each well, Lipofectamine RNAiMAX (Invitrogen) was used following the manufacturer’s instructions, with 50 nM of siRNA (si*Trpml1*, si*Ctsb*) added to each well.

### Microscale thermophoresis assay

The Monolith NT.115 instrument (NanoTemper Technologies) was used to measure the KD value. A range of concentrations of apilimod (from 0.4 mM to 200 nM) were incubated with 20 μg of purified His-GFP-NLRP3 protein for 40 min in assay buffer (50 mM Hepes, 10 mM MgCl2, 100 mM NaCl, pH 7.5, and 0.05% Tween 20). The samples were loaded into the NanoTemper glass capillaries, and MST was performed using 100% LED power and 80% MST power. The KD value was calculated using the mass action equation *via* the NanoTemper software from duplicate reads of an experiment.

### Statistical analyses

All values are expressed as the mean ± SEM. Statistical analysis was carried out using the unpaired *t*-test (GraphPad Software) with all data points showing a normal distribution. No exclusion of data points was used. The researchers were not blinded to the distribution of treatment groups during sample collection and data analysis. Sample sizes were selected on the basis of preliminary results to ensure an adequate power. Data were considered significant when *P*-values< 0.05.

## Results

### Apilimod promotes caspase-1 activation and IL-1β production in macrophages

**Figure 1:**
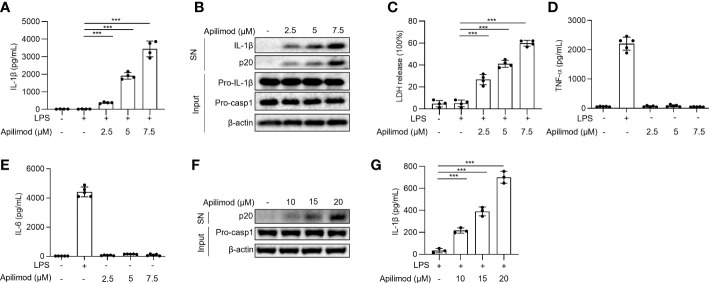
Apilimod induces caspase-1 activation and IL-1β secretion. **(A–E)** BMDMs were first primed with LPS for 3 h, then stimulated with doses of apilimod (2.5-7.5 μM) for 2 hours. The supernatant of the cell culture was collected. **(A)** ELISA of IL-1β releases in culture supernatants. **(B)** Immunoblot analysis of Active IL-1β and p20 (cleaved caspase-1) in supernatants (SN) and pro-IL-1β, pro-caspase-1 (pro-casp1) and *β*-actin in cell lysates (Input). **(C)** The release of LDH in supernatants **(D)** TNF-α and **(E)** IL-6 secretion levels in supernatants were detected by ELISA. **(F)** Immunoblot analysis of cleaved IL-1β and caspase-1 (p20) in culture supernatants (SN) and inactive precursor molecule (pro– IL-1β, pro–caspase-1(pro-casp1) in cell lysates (Input) of PMA-differentiated THP-1 cells stimulated with different doses of apilimod. **(G)** IL-1β secretion levels of PBMCs in supernatants were determined by ELISA. The data are from three independent experiments with biological duplicates in each and are shown as the mean ± SEM values (n = 4 or 3) or are representative of three independent experiments. One-way ANOVA was applied to calculate statistical significance: ***P < 0.001.

To investigate the mechanism of NLRP3 inflammasome activation, we used the small molecule library, to stimulate LPS-primed BMDMs for 2 hours, and detected IL-1β concentration in supernatants by ELISA. By library screening, we found that apilimod stimuli induces the secretion of IL-1β (data not shown). To investigate whether apilimod could induce the activation of inflammasome, mouse bone marrow-derived macrophages (BMDMs) were primed with LPS for 3 hours and then treated with apilimod for 2 hours. Apilimod dose-dependently induced IL-1β secretion and caspase-1 cleavage at concentrations of 2.5-7.5 μM (**Figures 1A, B**). It has been reported that apilimod stimulates lysosomal enlargement and B-lymphoma toxicity at lower concentrations. Our results showed that low concentration of apilimod enhances IL-1β secretion induced by nigericin, but cannot activate inflammasome (**Figures S1A, B**). When stimulating BMDMs with apilimod at 17.5 μM, the release of IL-1β reaches a maximum (**Figure S1C**). Further, apilimod provoked the release of lactate dehydrogenase (LDH) (**Figure 1C**), but had no effect on the production of TNF-α and IL-6 induced by LPS (**Figures 1D, E**). We also stimulated human peripheral blood mononuclear cells (PBMCs) with apilimod. The release of IL-1β and the activation of caspase-1 were detected after treatment with apilimod in a dose-dependent manner (**Figures 1F, G**). Taken together, our results confirmed that apilimod can induce caspase-1 activation and IL-1β production in murine and human macrophages.

### Apilimod activates the NLRP3 inflammasome

**Figure 2:**
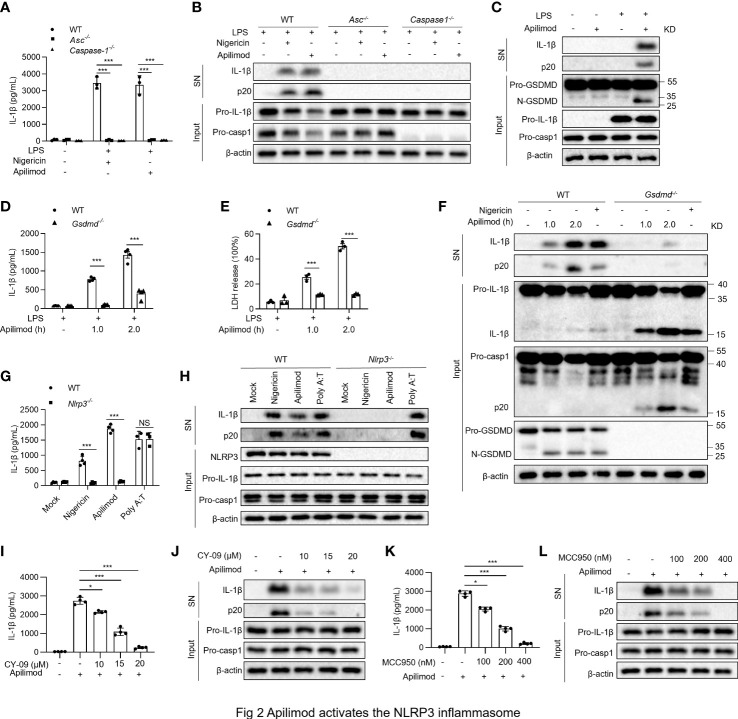
Apilimod triggers the activation of NLRP3 inflammasome. **(A, B)** BMDM cells of wild-type (WT) or *caspase1^-/-^* or *Asc^-/-^* mice were primed with LPS for 3 hours and then stimulated with apilimod (7.5 μM) for 2 hours. The supernatant of the cell culture was collected. **(A)** ELISA of IL-1β in the supernatant. **(B)** Western blot of IL-1β, cleaved caspase-1 in SN and pro-IL-1β, pro-caspase-1 (pro-casp1), β–actin in cell lysate (Input). **(C)** LPS-primed BMDM cells of wild-type (WT) mice were stimulated with apilimod (7.5 μM) for 2 hours. Western blot of IL-1β, cleaved caspase-1 in SN and cleaved GSDMD, pro-IL-1β, pro-caspase-1(pro-casp1), β–actin in cell lysate (Input). **(D–F)** LPS-primed BMDM cells of wild-type (WT) or *GSDMD* ^-/-^ mice were stimulated with apilimod (7.5 μM). **(D)** ELISA of IL-1β in the supernatant. **(E)** The release of LDH in supernatants **(F)** Western blot of IL-1β, cleaved caspase-1 in SN and pro-IL-1β, IL-1β, pro-caspase-1(pro-casp1), cleaved caspase-1, GSDMD, cleaved GSDMD, β–actin in cell lysate (Input). **(G, H)** LPS-primed BMDM cells of wild-type (WT) or *Nlrp3* ^-/-^ mice were stimulated with apilimod (7.5 μM) for 2 hours. **(G)** ELISA of IL-1β in the supernatant. **(H)** Western blot of IL-1β, cleaved caspase-1 in SN and pro-IL-1β, pro-caspase-1(pro-casp1), β–actin in cell lysate. **(I–L)** LPS-primed BMDMs were pretreated with CY-09 or MCC950 at different concentrations for 30 min and then stimulated with apilimod (7.5 μM) for 2 hours. **(I, K)** ELISA of mature IL-1β in the supernatants of BMDMs. **(J, L)** Western blot analysis of mature IL-1β and cleaved caspase-1 (p20) in supernatants (SN) of BMDMs and of pro-IL-1β and pro-caspase-1(pro-casp1) in the lysates (Input) of BMDMs. The data are from three independent experiments with biological duplicates in each and are shown as the mean ± SEM values (n = 3 or 4) or are representative of three independent experiments. One-way ANOVA or One-way ANOVA was applied to calculate statistical significance: *P < 0.05; ***P < 0.001; NS not significant.

To find out the pathway of caspase-1 activation and IL-1β production induced by apilimod, we stimulated BMDMs from wild-type (WT), *Asc^−/−^* and *Caspase-1^−/−^* mice with apilimod. IL-1β release and caspase-1 activation induced by apilimod are totally abrogated in *Asc^−/−^* and *Caspase-1^−/−^* macrophages (**Figures 2A, B**). Treatment with apilimod promoted the cleavage of GSDMD in BMDMs (**Figure 2C**), which leads to the formation of pores on cell membrane and pyroptosis. In *Gsdmd^-/-^* BMDMs, maturation IL-1β and p20 were trapped in the cells, and LDH release was also inhibited (**Figures 2D–F**). Thus, Apilimod-induced IL-1β secretion and cell death are dependent on GSDMD. Inflammasome activation is initiated by different kinds of cytosolic pattern recognition receptors (PRRs) that respond to various PAMPs or DAMPs. In order to identify the target of apilimod, we isolated BMDM cells from WT, *Nlrp3^-/-^*, *Aim2^-/-^* and *Pyrin^-/-^* mice, then stimulated with apilimod under the same conditions. The secretion of IL-1β induced by apilimod was comparable in WT, *Aim2^-/-^* and *Pyrin^-/-^* (**Supplementary Figures S2A, B**) BMDMs, but not in NLRP3-deficient cells (**Figure 2G**). Besides, the cleavage of caspase-1 was also blocked in NLRP3-deficient cells (**Figure 2H**). To validate the role of apilimod in NLRP3 inflammasome activation, we pretreated WT BMDMs with specific NLRP3 inflammasome inhibitors CY-09 (44) or MCC950 (45) respectively, and then stimulated with apilimod. The data showed that both CY-09 (**Figures 2I, J**) and MCC950 (**Figures 2K, L**) could inhibit apilimod-induced inflammasome activation in a dose-dependent manner. Taken together, these results indicated that apilimod specifically activates the NLRP3 inflammasome.

### Apilimod promotes the assembly of NLRP3 inflammasome

**Figure 3:**
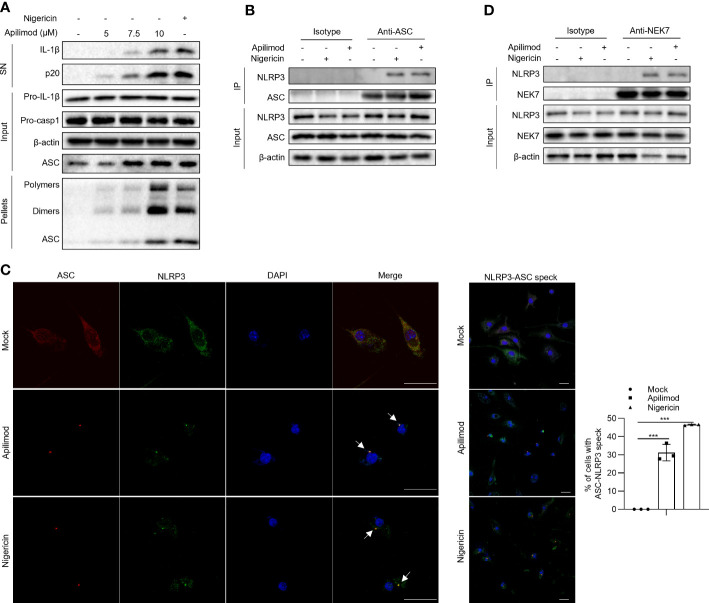
Apilimod promotes the assembly of NLRP3 inflammasome. **(A)** LPS-primed BMDMs were stimulated with apilimod at different concentrations for 2 hours, then lysed using TBS buffer. The TBS-insoluble pellet of cells was cross-linked with DSS at room temperature for 30 minutes. Crosslinked ASCs were detected by western blot. **(B, D)** Endogenous immunoprecipitation (IP) and western blot analysis to evaluate the NLRP3-ASC **(B)** interaction and NLRP3-NEK7 interaction **(D)** in LPS-primed BMDMs pretreated with apilimod for 2 h. **(C)** LPS-primed BMDMs were stimulated with apilimod (2 h) or nigericin (0.5 h). Representative immunofluorescence images displaying the subcellular distribution of NLRP3 (green) and ASC (red). Nuclei (blue) were revealed by DAPI. The merged images are displayed to demonstrate the co-localization of NLRP3 and ASC. The statistical graph represents the percentage of cells with ASC-NLRP3 speck. All data are representative of three independent experiments. One-wayANOVA or One-way ANOVA was applied to calculate statistical significance: ***P < 0.001.

To investigate the mechanism of apilimod-induced NLRP3 inflammasome activation, we assessed whether apilimod promotes the assembly of the NLRP3 inflammasome. It has been demonstrated that the ASC oligomerization is a critical step for the assembly of the NLRP3 inflammasome, which leads to the subsequent caspase-1 cleavage (46, 47). Through cross-linking experiments by disuccinimidyl suberate (DSS) and immunoblot assay, we found that apilimod induces ASC oligomerization in a concentration-dependent manner (**Figure 3A**), suggesting that apilimod promotes the NLRP3 inflammasome activation upstream of ASC oligomerization. The interaction between NLRP3 and ASC is essential for the recruitment of ASC and subsequent ASC oligomerization (1, 48). We performed a co-immunoprecipitation experiment, and the results showed that apilimod considerably increases the endogenous NLRP3-ASC interaction (**Figure 3B**). Microscopy images showed that apilimod promotes the formation of ASC-NLRP3 speck, similarly to cells treated with nigericin (**Figure 3C**). In addition to the interaction between NLRP3 and ASC, the NLRP3-NEK7 interaction also plays a vital role in the upstream of NLRP3 inflammasome activation (49). Apilimod also facilitates the endogenous interaction between NLRP3 and NEK7 (**Figure 3D**). All of the above results indicated that apilimod induces the activation of NLRP3 inflammasome by promoting the assembly of the complex.

### NLRP3 inflammasome activation induced by apilimod is independent of direct binding and potassium efflux

**Figure 4:**
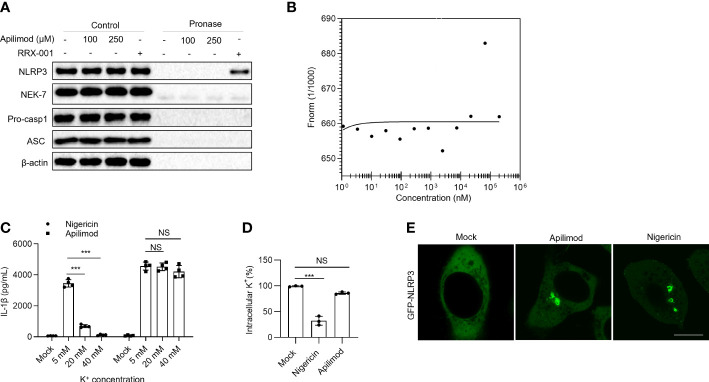
Direct binding and ion flow are not required for NLRP3 inflammasome activation by apilimod. **(A)** LPS-primed BMDMs cell lysates incubated with indicated doses of apilimod, and then digested with pronase (25 ng/μg of protein), NLRP3, NEK7, pro-caspase-1 (pro-casp1), ASC and *β*-actin were detected by western blot. **(B)** Binding affinity was analyzed by MST between apilimod and purified GFP-NLRP3 protein. HEK-293T cells transfected with Flag-tagged NLRP3. **(C)** LPS-primed BMDMs were stimulated with nigericin (5 µM), apilimod (7.5 μM) in medium containing different concentrations of K^+^ and secreted IL-1β was measured by ELISA. **(D)** ICP of intracellular potassium in LPS-primed BMDM cells of *Nlrp3* ^-/-^ mice stimulated with apilimod (7.5 μM) for 2 hours or nigericin (5 μM) for 20 minutes. **(E)** Analysis by confocal microscopy of HeLa cells stably expressing NLRP3-GFP were stimulated with apilimod or nigericin. The data are from three independent experiments with biological duplicates in each and are shown as the mean ± SEM values (n = 3 or 4) or are representative of three independent experiments. One-way ANOVA or One-way ANOVA was applied to calculate statistical significance: ***P < 0.001; NS not significant.

The next step was to investigate the cellular mechanism by which apilimod activates the NLRP3 inflammasome. To detect whether apilimod binds NLRP3, we applied the drug affinity responsive target stability (DARTS) assay, which is predicated on the decrease in protease susceptibility of a target protein upon drug binding. BMDMs cell lysates were incubated with different concentrations of apilimod, then digested with the protease pronase (50). While RRx-001, that was reported to directly bind NLRP3, effectively inhibits NLRP3 degradation by pronase, apilimod is unable to prevent protease-mediated proteolysis of NLRP3 (**Figure 4A**). Microscale thermophoresis (MST) was utilized to further verify the direct contact between apilimod and purified GFP-NLRP3. According to the data, the incubation of apilimod with purified GFP-NLRP3 caused no alterations in fluorescence (**Figure 4B**), indicating that there is no direct binding between them. Thus, apilimod does not modulate the activation of NLRP3 *via* direct interaction.

Cytosolic potassium (K^+^) efflux has been proposed to be a common and important upstream event in NLRP3 inflammasome activation (7, 9). To detect whether apilimod affects the potassium efflux during NLRP3, we tested the activation of NLRP3 induced by apilimod and nigericin in medium with different K^+^ concentrations (5 mM, 20 mM, 40 mM). The results showed that high concentration of extracellular potassium ions significantly inhibits NLRP3 inflammasome activation induced by nigericin, but not apilimod (**Figure 4C**). We also measured cellular concentrations of potassium after apilimod and nigericin stimulation. Compared with the untreated control cells, the concentration of intracellular potassium was not altered by apilimod treatment, whereas it was substantially reduced after nigercin treatment (**Figure 4D**). In summary, the activation of the NLRP3 inflammasome induced by apilimod is independent of direct binding to NLRP3 and K^+^ efflux. The recruitment of NLRP3 to dTGN or endosomes has been reported to be an important upstream signal of NLRP3 activation. In Hela cells that stably expressed GFP-NLRP3, apilimod promoted NLRP3 redistribution, to form rings in cytoplasm, which is the same as nigericin treatment (**Figure 4E**).

### Apilimod mediates NLRP3 inflammasome activation through mitochondrial damage and reactive oxygen species production

**Figure 5:**
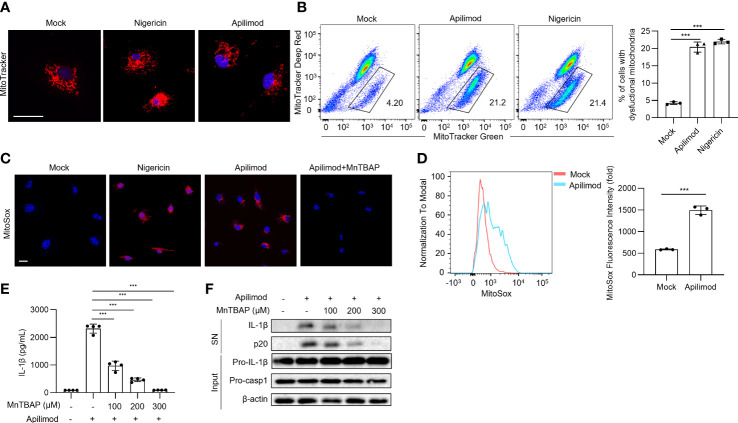
Apilimod mediates inflammasome activation through mitochondrial damage and reactive oxygen species production. **(A)** Analysis by confocal microscopy of LPS-primed BMDMs stimulated with nigericin or apilimod and subsequently stained with MitoTracker Red (50 nM) follow by the DNA-binding dye DAPI (blue). Scale bar, 20 μm. **(B)** Analysis by FACS of THP1 cells stimulated with nigericin or apilimod and subsequently stained with MitoTracker deep red (50 nM) and MitoTracker green (50 nM). **(C, D)** Analysis by confocal microscopy and FACS of BMDMs stimulated with nigericin or apilimod and subsequently stained with MitoSOX Red (1 μM). Scale bar, 20 μm. **(E, F)** LPS-primed BMDMs were pretreated with a ROS inhibitor (MnTBAP, 150 μM) for 30 min and then stimulated with apilimod for 2 hours. **(E)** ELISA of IL-1β in culture supernatants. **(F)** Western blot of IL-1β, cleaved caspase-1 in supernatants (SN) and pro-IL-1β, pro-caspase-1, β–actin in cell lysate (Input). The data are from three independent experiments with biological duplicates in each and are shown as the mean ± SEM values (n = 3 or 4) or are representative of three independent experiments. One-way ANOVA was applied to calculate statistical significance: ***P < 0.001.

Damaged mitochondria and their released mitochondrial signals, such as mitochondrial DNA (mtDNA) and mitochondrial reactive oxygen species (mtROS), are considered upstream events that contribute to NLRP3 inflammasome activation (14–16). To verify whether apilimod induces NLRP3 activation through mitochondrial damage, we detected the mitochondrial status of BMDMs stained with MitoTracker Red dye after apilimod stimulation, and observed abnormal mitochondrial fission, which changed from normal scattered lines to broken points or rods (**Figure 5A**). In *Nlrp3^-/-^* cells, apilimod also induces mitochondrial damage and ROS production, indicating that mitochondrial dysfunction induced by apilimod is upstream of NLRP3 activation (**Figures S3A, B**). Meanwhile, apilimod stimulation induces the loss of mitochondrial membrane potential, determined by dyeing respiring and total mitochondria (**Figure 5B**). In accordance with this, mitochondrial reactive oxygen species (ROS) production was enhanced by apilimod treatment (**Figures 5C, D**). MnTBAP is a cell-permeable synthetic antioxidant with superoxide dismutase-like activity, which can scavenge ROS effectively in the cytoplasm (51). We pretreated LPS-primed bone marrow-derived macrophages (BMDMs) with MnTBAP for 30 min and then stimulated the cells with apilimod. MnTBAP obviously inhibited mitochondrial ROS production induced by apilimod (**Figure 5C**). IL-1β secretion and caspase-1 cleavage induced by apilimod were blocked by MnTBAP, which is dose-dependent (**Figures 5E, F**). These results suggested that apilimod activates the NLRP3 inflammasome in a mitochondrial-dependent manner.

### Apilimod induces mitochondria damage and NLRP3 activation through TRPML1-dependent lysosomal calcium flux

To delineate the upstream mechanisms involved in apilimod-mitochondria damage induced NLRP3 inflammasome activation, we first determined whether apilimod impairs the mitochondria-lysosome interaction which dynamically regulates mitochondrial fission. Previous study has reported that the contact formation is promoted by active GTP-bound lysosomal RAB7 (52). We pretreated LPS-primed BMDM cells with CID1067700, a late endosome GTPase RAB7 receptor antagonist (53), and then stimulated with apilimod. The result showed that CID1067700 does not inhibit the secretion of IL-1β induced by apilimod (**Figure S4A**), demonstrating that apilimod-induced mitochondrial damage was not caused by direct contact with lysosomes.

Apilimod can lead to lysosomal dysfunction (43), which has been reported to play an important role in the activation of NLRP3 inflammasome (11, 12). Inhibition of lysosomal acidification and cathepsin B have been reported to impair NLRP3 activation. Our results showed that the mean fluorescence intensity of Lysotracker green was significantly increased after apilimod stimulation (**Figures S4B–D**). Bafilomycin A, a blocker of the vascular H^+^ ATPase system (54), significantly inhibited the lysosomal acidification induced by apilimod (**Figures S4B–D**), and apilimod-induced NLRP3 inflammasome activation (**Figures S4E, F**). Cathepsin B inhibitor CA-074-Me treatment also inhibits apilimod-induced NLRP3 activation (**Figures S4G, H**). Furthermore, bafilomycin A and CA-074-Me inhibited apilimod-stimulated mitochondrial damage (**Figures S4I, J**). However, cathepsin B knockdown had no effect on apilimod-induced inflammasome activation (**Figure S4K**), indicating that the inhibitor might has off-target effects.

**Figure 6:**
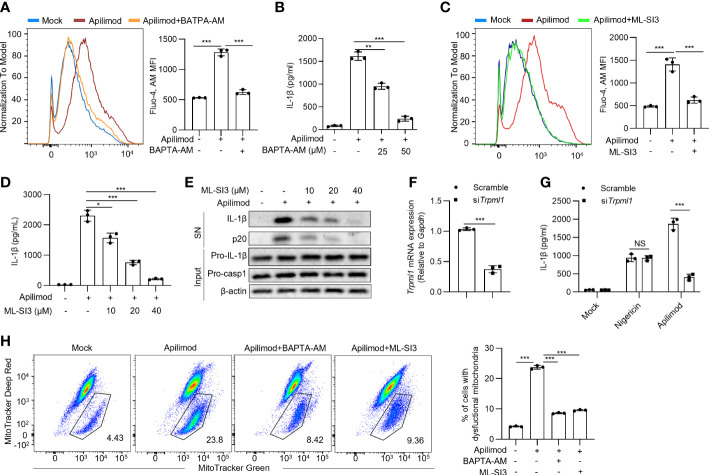
Apilimod damage the mitochondria through inducing lysosomal calcium flux. **(A, B)** BMDMs were first primed with LPS for 3 hours, then pretreated with BAPTA-AM (25 μM, 50 μM) for 30 minutes, and stimulated with apilimod for 2 hours. **(A)** Fluo-4 AM (5 μM) staining of BMDMs after apilimod treatment, FACS analysis was conducted to show changes in cell calcium flux. **(B)** ELISA of IL-1β in culture supernatants. **(C–E)** LPS-primed BMDMs pretreated with ML-SI3 for 30 min and then stimulated with apilimod. **(C)** Fluo-4 AM staining of BMDMs after apilimod treatment, FACS analysis was conducted to show changes in cell calcium flux. **(D)** ELISA of IL-1β in culture supernatants.**(E)** Western blot of IL-1β, cleaved caspase-1 in supernatants (SN) and pro-IL-1β, pro-caspase-1 (pro-casp1), β–actin in cell lysate. **(F, G)** BMDMs transfected with siRNA against *Trpml1* or control siRNA were primed with LPS for 3 h and were then treated with apilimod or nigericin. The supernatant of the cell culture was collected. **(F)** *Trpml1* mRNA in BMDMs. **(G)** ELISA of IL-1β in the supernatants of BMDMs. **(H)** LPS-primed THP-1 cells were pretreated with BAPTA-AM (50 μM) or ML-SI3 (40 μM) for 30 minutes, and stimulated with apilimod for 2 hours. subsequently stained with MitoTracker deep red (50 nM) and MitoTracker green (50 nM). Analysis by FACS of THP1 cells. The data are from three independent experiments with biological duplicates in each and are shown as the mean ± SEM values (n = 3) or are representative of three independent experiments. One-way ANOVA was applied to calculate statistical significance: **P < 0.01; ***P < 0.001.

The other pathway is that phagocyted contents or agonists targeting lysosomes cause the ion flux of lysosomes to activate the NLRP3 inflammasome. We found that apilimod treatment increases cellular calcium, as observed by staining with Fluo-4, AM probe (**Figure 6A**). To investigate the role of calcium in apilimod-induced inflammasome activation, we used the calcium chelator BAPTA-AM to treat BMDMs before apilimod stimulation. BATPA was able to inhibit apilimod-induced cellular calcium. Meanwhile, BAPTA-AM dose-dependently inhibited apilimod-induced NLRP3 inflammasome activation (**Figure 6B**). Thus, NLRP3 activation triggered by apilimod is dependent on cellular calcium. Next, we used 2-APB, an antagonist of endoplasmic reticulum calcium channel protein, to pretreat cells before apilimod stimulation. The results showed that inhibition of ER calcium flux has no effect on inflammasome activation induced by apilimod (**Figure S5A**), indicating that apilimod-induced NLRP3 activation is independent of ER calcium flux. ML-SI3 is an inhibitor of TRPML1, a calcium channel protein on the lysosome membrane. ML-SI3 could inhibit apilimod-induced cellular calcium increase (**Figure 6C**). Besides, ML-SI3 inhibited the NLRP3 inflammasome activation induced by apilimod (**Figures 6D, E**). Consistent with previous reports, BAPTA-AM could inhibit nigericin-mediated calcium efflux and IL-1β production (**Figures S6A, B**), whereas ML-SI3 could not inhibit the above processes (**Figures S5B, C**).

While TRPML1 knockdown had no effects on nigericin-induced NLRP3 activation, it significantly inhibited apilimod-induced IL-1β secretion (**Figures 6F, G**). These data indicated that apilimod activates the NLRP3 inflammasome by inducing lysosomal calcium flux. Further, inhibition of calcium flux or lysosomal calcium channel abrogated mitochondrial damage triggered by apilimod (**Figure 6H**), while the inhibition of lysosomal calcium channel did not eliminate mitochondrial damage induced by nigericin (**Figure S6C**). Apilimod suppresses the forward trafficking of endosomes to fusion with lysosomes, which results in a massive accumulation of endosomes which coalesce into large vacuoles (55). During apilimod-induced NLRP3 activation, apilimod also induced the formation of large vacuoles in macrophages. Bafilomycin A1 and calcium inhibition by BAPTA-AM could inhibit both NLRP3 activation and vacuoles induced by apilimod. ML-SI3 can effectively inhibit apilimod-induced NLRP3 activation, but cannot affect vacuoles formation. Therefore, vacuoles formation triggered by apilimod is not necessary for NLRP3 inflammasome activation (**Figure S7**). Collectively, apilimod promotes calcium flux in lysosomes to induce mitochondrial damage and following NLRP3 inflammasome activation.

## Discussion

In this study, we found that aplimod, an inhibitor of the interleukins IL-12 and IL-23 production, can specifically activate the NLRP3 inflammasome. Apilimod triggers the NLRP3 inflammasome activation in a lysosomal-calcium-mitochondria dependent manner. We discovered apilimod as a novel NLRP3 inflammasome activator and investigated the cellular mechanism for apilimod activating NLRP3. Apilimod has the potent to be a molecular probe to help explore the mechanism of NLRP3 inflammasome activation. NLRP3 can be activated by various agonists, including ATP, nigericin, MSU crystals, alum, etc. Among them, many activators induce K^+^ efflux upstream of NLRP3 inflammasome assembly. And high concentration of extracellular K^+^ were reported to inhibit NLRP3 activation. However, recent research found several agonists activate NLRP3 without altering potassium flux. The TLR7 ligand imiquimod and its derivative CL097 bypass induction of potassium efflux and mediate NLRP3 inflammasome activation through directly acting on mitochondria. Imiquimod and CL097 target to quinone oxidoreductases NQO2 and mitochondrial complex I to induce ROS production, leading to NEK7-NLRP3 assembly to form the inflammasome complex. In our findings, apilimod is also a K^+^-independent NLRP3 agonist. But it targets to lysosomes to induce calcium flux-mediated mitochondrial damage, which is different from imiquimod and CL097.

Lysosomes are essential cellular organelles for digesting numerous pathogenic or toxic chemicals absorbed from the environment (20, 22, 23). The autophagosome frequently fuses with the lysosome, allowing the degradation of its contents by a series of enzymes capable of digesting all biological polymers (proteins, nucleic acids, and lipids). Either way, lysosomes can be damaged at times, when the cell engulfs particles that are too large or otherwise too difficult to degrade. This results in the release of their contents into the surrounding environment. Monosodium urate crystals, alum, and silica have been reported to cause lysosomal disruption and the NLRP3 inflammasome activation (11, 12, 29), but how lysosomes mediate the NLRP3 activation is unknown. In this investigation, we discovered that apilimod triggers mitochondrial damage in a lysosomal-dependent way. Apilimod induces calcium release from lysosomes. Cellular calcium chelating agent and lysosome calcium channel inhibitor are able to abrogate apilimod-induced mitochondrial damage and NLRP3 inflammasome activation. Thus, our results indicate the mechanism that apilimod activates NLRP3 through a lysosome-calcium-mitochondria pathway.

During the experiment, we noticed that apilimod altered the morphology of BMDMs. After 15-minute stimulation, dense vesicles appeared in the cells, which grew in size and would not vanish over time. The formation of vesicles might be caused by endosomal dysfunction, which then leads to lysosomal dysfunction, as established by a review of pertinent literature (54). Bafilomycin A1 and BAPTA-AM can significantly inhibit both the formation of vacuoles and the production of inflammasomes. However, while lysosomal calcium channel inhibitor abrogated NLRP3 inflammasome activation induced by apilimod, it could not inhibit vacuoles formation, indicating that vacuoles formation is not necessary in NLRP3 activation.

Apilimod was previously discovered to be an inhibitor of Toll-like receptor-induced IL-12 and IL-23 cytokine production, and it was tested in clinical trials as an immunomodulatory agent for the treatment of T helper 1 (Th1)- and Th17-mediated inflammatory diseases. In these trials, healthy volunteers as well as people with psoriasis, rheumatoid arthritis, and Crohn’s disease were included. Apilimod induced mild to moderate adverse reactions in treated patients. In phase II clinical trial, inflammatory disease indications for apilimod did not achieve the primary end goals, and further clinical research was stopped (38–41). In this study, we found that apilimod could promote IL-1β secretion and caspase-1 cleavage in BMDM cells and human peripheral blood mononuclear cells (PBMCs). And low concentration of apilimod enhances NLRP3 inflammasome activation. The pro-inflammatory function of apilimod may explain the side effects and ineffective treatment outcome in phase II clinical trials.

Collectively, we discovered that apilimod is a new specific agonist of the NLRP3 inflammasome. Subsequently, we will utilize mass spectrometry and CRISPR-Cas9 screening techniques to detect changes in small molecules and proteins in cells treated by apilimod, to identify the key upstream signals of NLRP3 inflammasome activation.

## Data availability statement

The raw data supporting the conclusions of this article will be made available by the authors, without undue reservation.

## Ethics statement

The animal study was reviewed and approved by Institutional Animal Care of the University of Science and Technology of China.

## Author contributions

YH and HH performed the experiments of this work. RZ designed the research. YH, MM, and RZ wrote the manuscript. RZ supervised the project. All authors contributed to the article and approved the submitted version.
