# Supplementary material for: Apilimod activates the NLRP3 inflammasome through lysosome-mediated mitochondrial damage
Source: Front Immunol. 2023 Jun 8;14:1128700. doi: 10.3389/fimmu.2023.1128700 (PMC10285205; doi:10.3389/fimmu.2023.1128700)
Supplement: Supplementary file 1 [file DataSheet_1.docx]

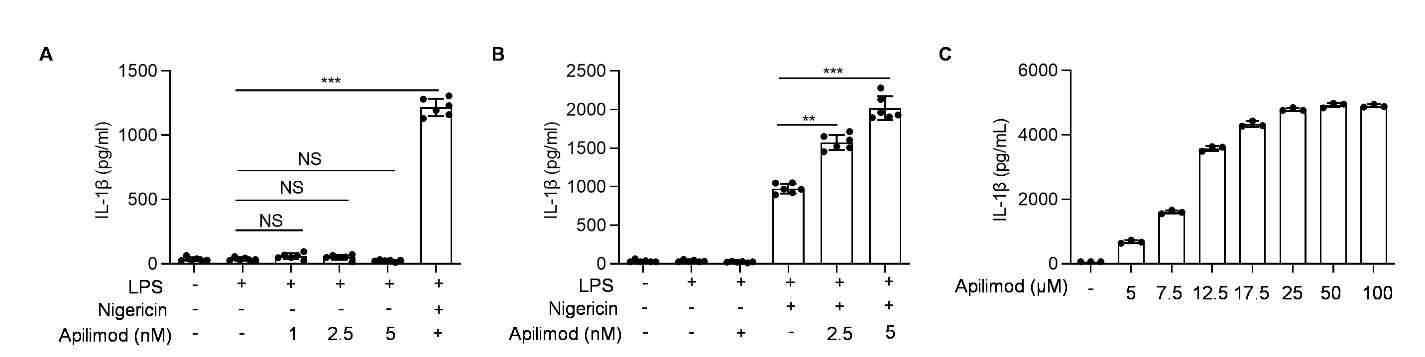


**Supplementary Figure 1 The effect of different concentrations of apilimod on the activation of inflammasome.** (**A**) LPS-primed BMDMs were sitmulated with apilimod (1 nM, 2.5 nM, 5 nM) for 2 hours, the cell supernatant was collected for ELISA analysis of IL-1β levels. (**B**) BMDMs were pretreated with apilimod (2.5 nM, 5 nM) for 12 hours, then stimulated with nigericin (5 μM) for 0.5 hours, the cell supernatant was collected for ELISA analysis of IL-1β levels. (**C**) LPS-primed BMDMs were sitmulated with different concentrations of apilimod (5 μM-100μM) for 2 hours, the cell supernatant was collected for ELISA analysis of IL-1β levels.. All data are representative of three independent experiments.


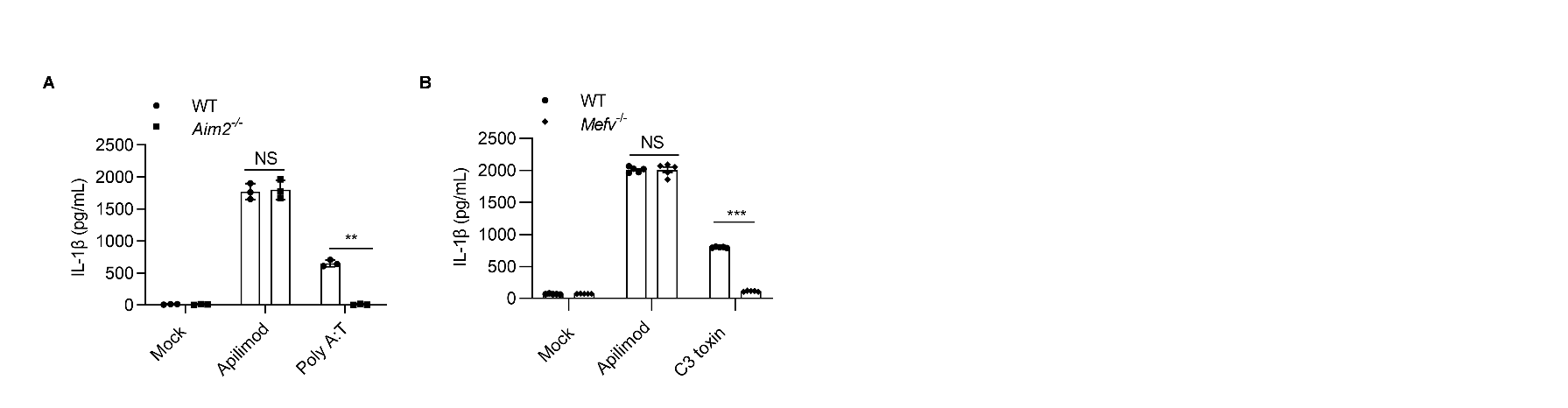


**Supplementary Figure 2 Apilimod-induced caspase-1 activation and IL-1β production is independent of AIM2 or Pyrin inflammasome.** **(A)** LPS-primed BMDM cells of wild-type (WT) or *Aim2*^-/-^ mice were stimulated with apilimod (7.5 μM), nigericin (5 μM), polyA:T. The supernatant of the cell culture was collected and subjected to ELISA to quantify the levels of IL-1β. **(B)** LPS-primed BMDM cells of WT or *Pyrin*^-/-^ (*Mefv^-/-^*) mice were stimulated with apilimod (7.5 μM), nigericin (5 μM), C3 toxin. The supernatant of the cell culture was collected and subjected to ELISA to quantify the levels of IL-1β. The data are from three independent experiments with biological duplicates in each and are shown as the mean ± SEM values (n = 3 or 4). Two-way ANOVA was applied to calculate statistical significance: ***P* < 0.01; ****P* < 0.001; NS not significant.

**
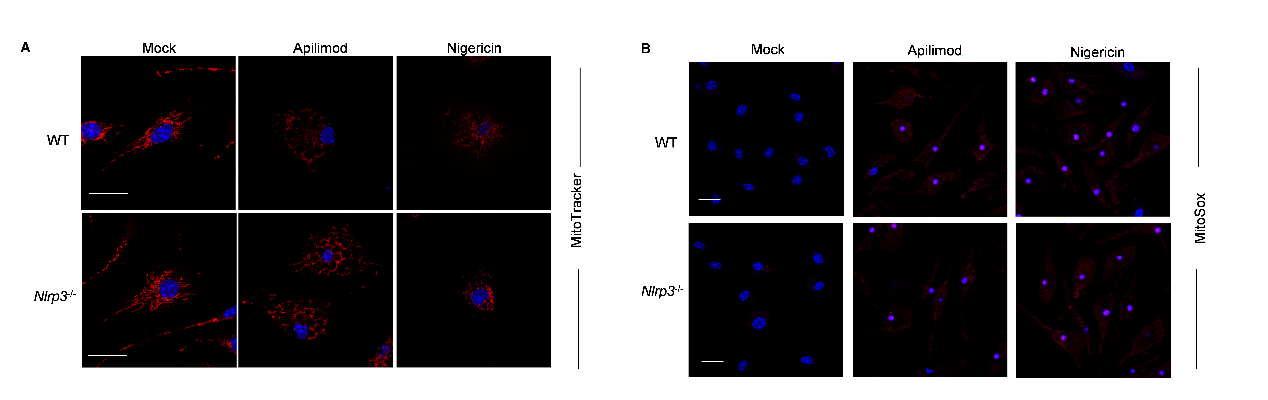
**

**Supplementary Figure 3 Mitochondrial dysfunction induced by apilimod is upstream of NLRP3 activation.(A, B)** BMDM cells of wild-type (WT) or *Nlrp3^-/-^* mice were primed with LPS for 3 hours, followed by stimulation with apilimod (7.5 μM) for 2 hours. Analysis by confocal microscopy of LPS-primed BMDMs stained with MitoTracker Red(**A**) or MitoSOX Red(**B**) follow by the DNA-binding dye DAPI (blue). Scale bar, 20 μm. All data are representative of three independent experiments.


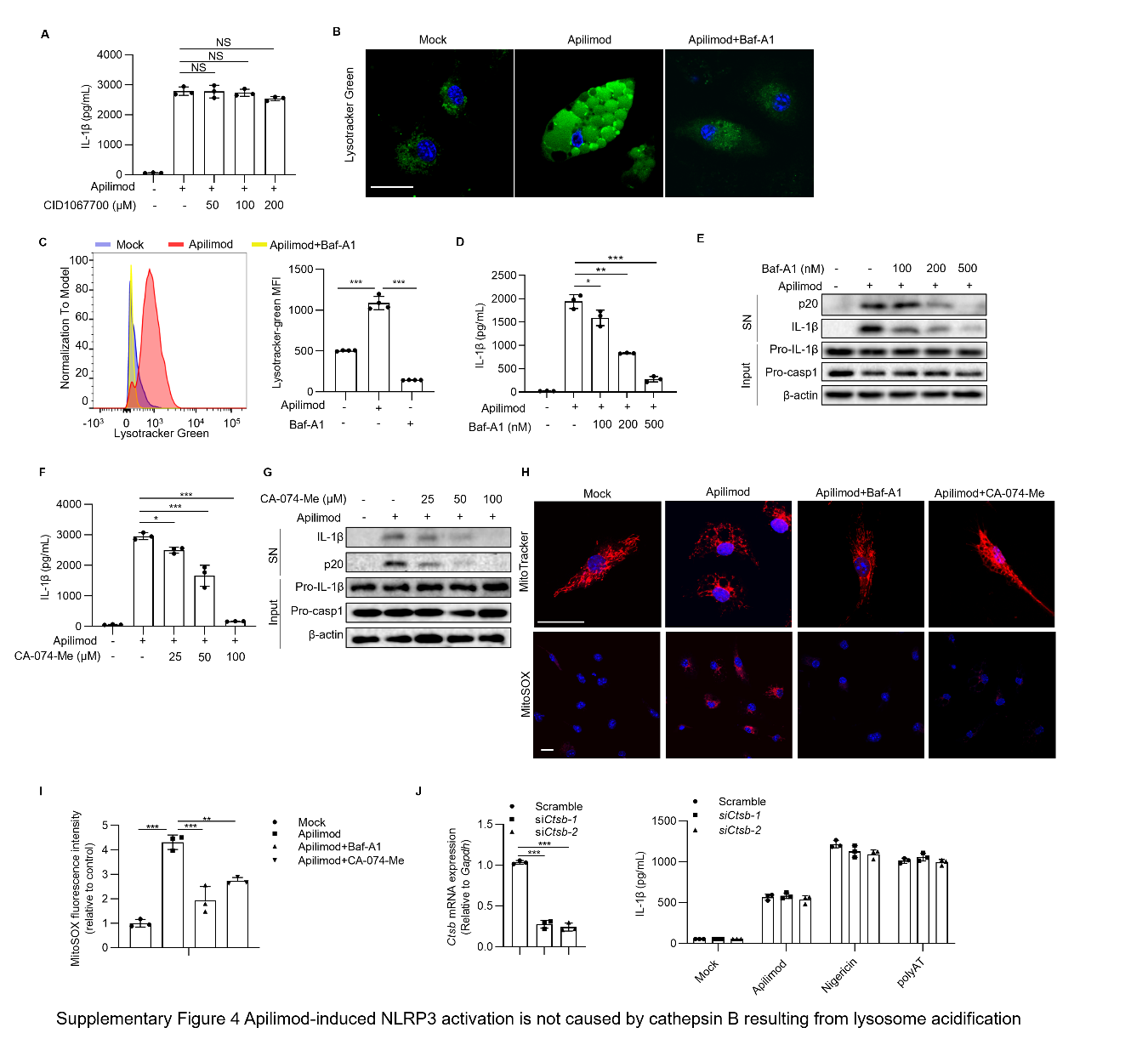


**Supplementary Figure 4** **Apilimod induces inflammasome activation is independ of Cathepsin B resulting from lysosomal acidification.** **(A)** LPS-primed BMDMs were pretreated with CID1067700 at different concentrations for 30 min and then stimulated with apilimod for 2 hours. IL-1β levels in culture supernatants were measured by ELISA. (**B, C**) LPS-primed BMDMs were pretreated with with or without Bafilomycin A1(lysosomal acidification inhibitor), then stimulated with apilimod and stained with Lysotracker Green (100 nM) following by the DNA-binding dye Hoechst 33342 (blue). (**B**) Analysis by confocal microscopy. Scale bar, 20 μm. (**C**) Analysis by FACS, a representative histogram and quantification of the results is shown (n= 4 ). (**D-E**) LPS-primed BMDMs were pretreated with bafilomycin A1 at different concentrations for 30 min and then stimulated with apilimod for 2 hours. (**D**) ELISA of IL-1β in culture supernatants. (**E**) Western blot of IL-1β, cleaved caspase-1 in supernatants (SN) and pro-IL-1β, pro-caspase-1 (pro-casp1), β–actin in cell lysate. (**F-G**) LPS-primed BMDMs were pretreated with CA-074-Me at different concentrations for 30 min and then stimulated with apilimod for 2 hours. (**F**) ELISA of IL-1β in culture supernatants. (**G**) Western blot of IL-1β, cleaved caspase-1 in supernatants (SN) and pro-IL-1β, pro-caspase-1 (pro-casp1), β–actin in cell lysate. (**H**) Analysis by confocal microscopy of LPS-primed BMDMs pretreated with or without Bafilomycin A1 or CA-074-Me, then stimulated with apilimod and subsequently stained with MitoTracker Red or MitoSOX Red follow by the DNA-binding dye DAPI (blue). Scale bar, 20 μm. (**I**) Mean Mito Sox fluorescence intensities are presented as percentage relative to the control value. (**J**) BMDMs transfected with siRNA against *Ctsb* or control siRNA were primed with LPS for 3 h and were then treated with apilimod or nigericin. *Ctsb* mRNA in BMDMs. ELISA of IL-1β in the supernatants of BMDMs. The data are from three independent experiments with biological duplicates in each and are shown as the mean ± SEM values (n = 4) or are representative of three independent experiments. One-way ANOVA was applied to calculate statistical significance: *P < 0.05; **P < 0.01; ***P < 0.001; NS not significant.


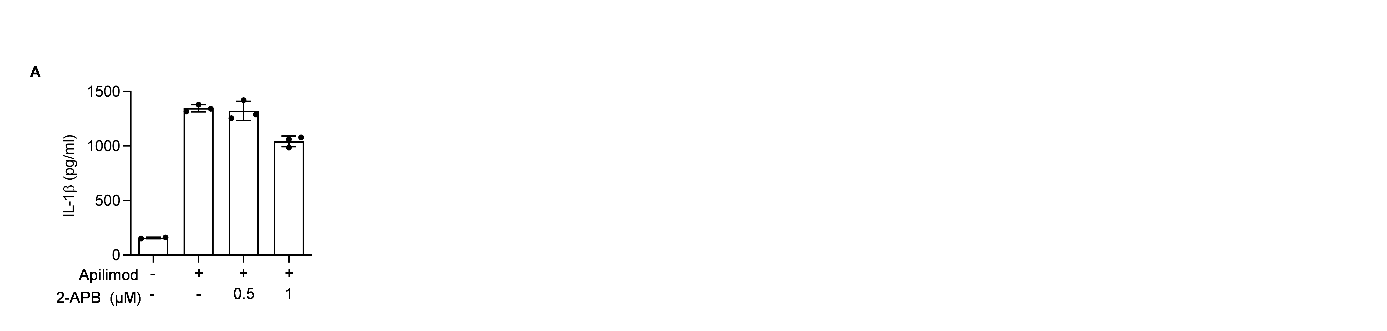


**Supplementary Figure 5 Apilimod-induced NLRP3 activation is independent of ER calcium flux. (A)** LPS-primed BMDMs were pretreated with 2-APB at different concentrations for 30 min and then stimulated with apilimod for 2 hours. The supernatant of the cell culture was collected and subjected to ELISA to quantify the levels of IL-1β.


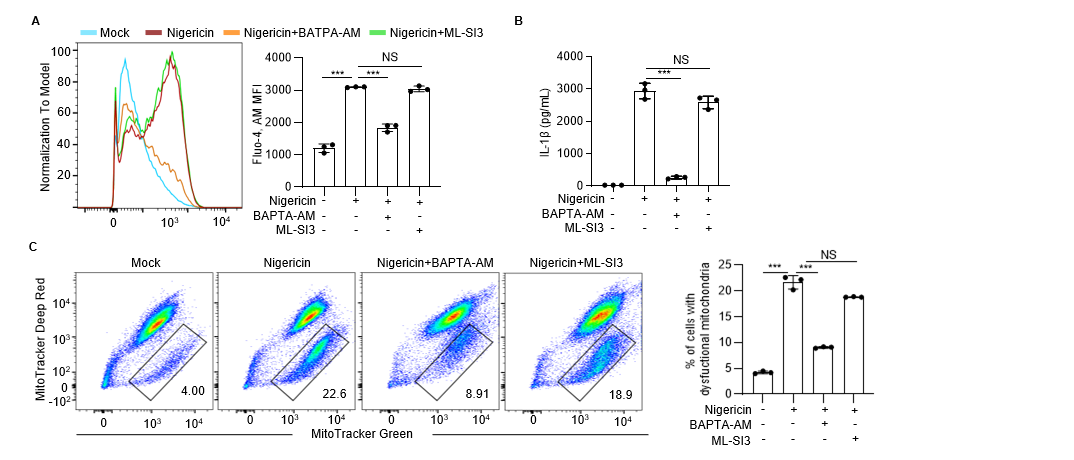


**Supplementary Figure 6 Nigericin-induced NLRP3 activation is independent of lysosomal calcium flux.** (**A-C**) LPS-primed BMDMs were pretreated with BAPTA-AM (50 μM) or ML-SI3 (40 μM) for 30 minutes, then stimulated with nigericin for 30 minutes. (A) Fluo-4 AM (5 μM) staining of BMDMs after apilimod treatment, FACS analysis was conducted to show changes in cell calcium flux. (**B**) ELISA of IL-1β in culture supernatants.(C) Analysis by FACS of THP1 cells after apilimod stimulation and subsequent staining with MitoTracker deep red (50 nM) and MitoTracker green (50 nM). Analysis by FACS of THP1 cells. The data are from three independent experiments with biological duplicates in each and are shown as the mean ± SEM values (n = 3) or are representative of three independent experiments. One-way ANOVA was applied to calculate statistical significance: ***P* < 0.01; ****P* < 0.001.


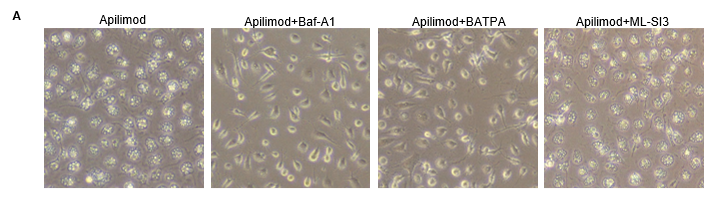


**Supplementary Figure 7 Vacuoles formation triggered by apilimod is not necessary in NLRP3 inflammasome activation.** (**A**) LPS-primed BMDMs were pretreated with Bafilomycin A1, BAPTA-AM, or ML-SI3 for 30 minutes, then stimulated with apilimod for 2 hours. Observe cell morphology and vacuole formation under a microscope.
